# Spontaneous vesicorectal fistula: a rare complication of neurogenic bladder

**DOI:** 10.1186/s12894-020-00585-7

**Published:** 2020-02-24

**Authors:** Weilin Fang, Lei Xu, Jiabei Sun, Jacques Corcos, Jiayi Li

**Affiliations:** 1 Department of Urology, Renji Hospital, School of Medicine, Shanghai Jiaotong University, Shanghai, 200127 People’s Republic of China; 2 Department of Urology, Jewish General Hospital, 3755 Côte Ste Catherine Road, Suite E-945, Montreal, QC Canada

**Keywords:** Vesicorectal fistula, Neurogenic bladder, Video urodynamic

## Abstract

**Background:**

We report a rare case of spontaneous vesicorectal fistula.

**Case presentation:**

A 13-year-old female spina bifida patient who complained of fecal and urinary incontinence was eventually diagnosed with a spontaneous vesicorectal fistula. We hypothesized that infection, neurogenic bowel and neurogenic bladder caused her vesicorectal fistula. The patient refused the operation, and she is currently in a delicate balance.

**Conclusions:**

Early repair of the fistula is necessary. The treatment of neurogenic bladder after fistula repair is difficult and deserves further observation and follow-up.

## Background

Myelomeningocele is frequently associated with neurogenic bladder dysfunction. In these cases, tethered cords are a common finding. Age and height growth may worsen tethered cord syndrome, including symptoms of bladder and bowel dysfunction, pain, and lower limb malformation. In advanced cases, bladder and upper urinary tract distension as well as rectal distension may lead to severe complications. We report a rare case of spontaneous vesicorectal fistula.

## Case presentation

In 2017, we managed a 13-year-old female spina bifida patient with fecal and urinary incontinence associated with recurrent urinary tract infections. Myelomeningocele repair was performed at 4 months after birth. At the age of 4, she began clean intermittent self-catheterization (CIC) due to weak bladder emptying and hydronephrosis. Further follow-up showed remission of hydronephrosis. Two years later, she stopped CIC on her own and was lost to follow-up. She is currently complaining of urinary incontinence, urinary dribbling, and stool incontinence.

**Fig. 1:**
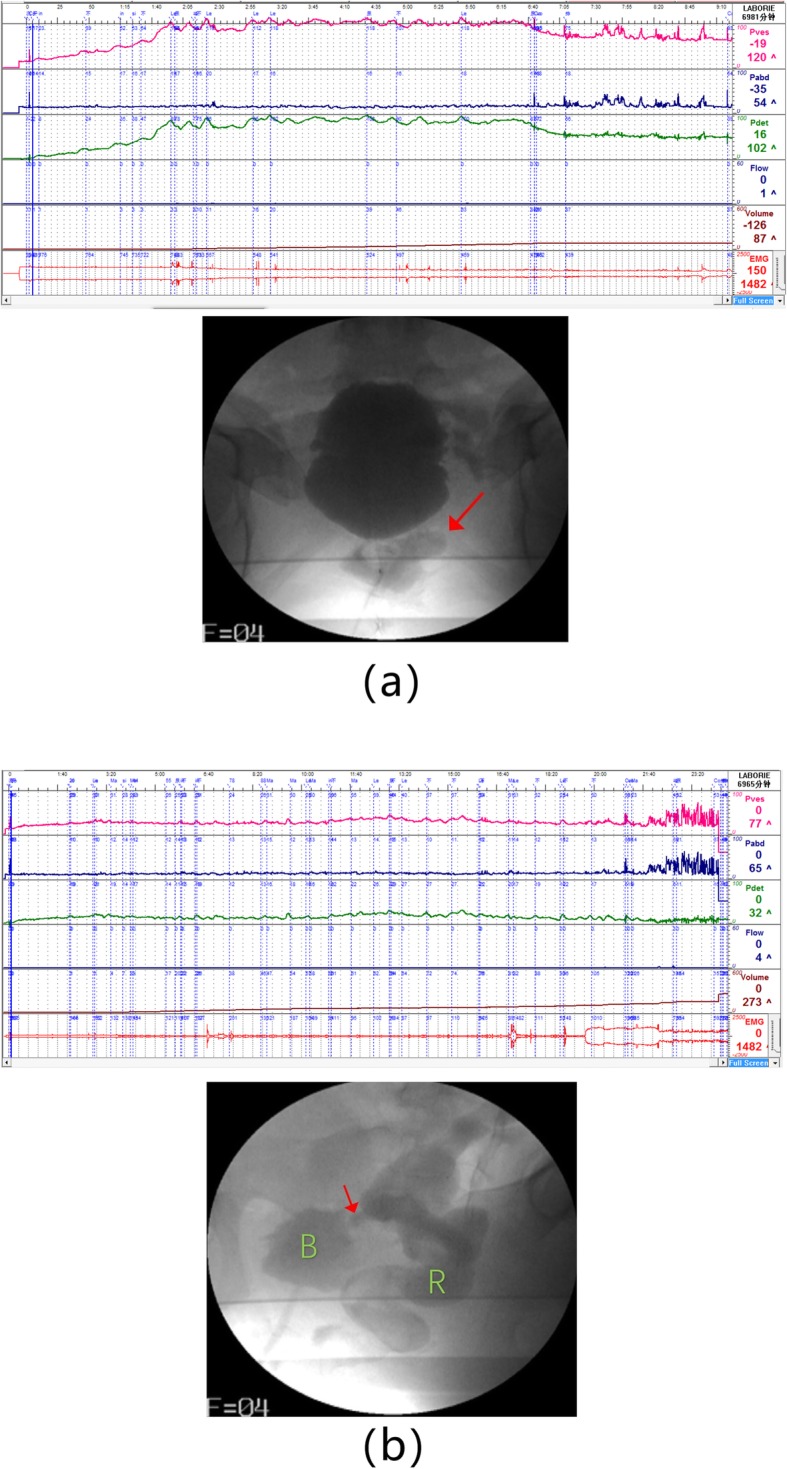
**a** Result and radiograph of patient’s first video urodynamic. Bladder compliance significantly decreased during the filling phase(C = 2.12 ml/cmH2O). In the anteroposterior film, there is a suspicious concentration of contrast around the bladder (arrow). **b** Result and radiograph of patient’s second video urodynamic. Bladder compliance was normal this time (C = 31). On oblique incidences, the contrast agent leaked into rectum from the bladder (B: bladder; R: rectum; arrow: fistula)

Her postvoid residual urine and upper urinary tract ultrasound were normal. We performed a video urodynamic study (Fig. 1 (a) and Table 1). It showed decreased bladder compliance (C = 2.12 ml/cmH_2_O) and detrusor overactivity with no ureteral reflux. There was no detrusor contraction during the voiding phase, but abdominal straining assisted voiding. There was no voluntary voiding (most urine leaked slowly during the filling phase). At the end of the voiding phase, we noticed a concentration of contrast around the bladder. To explain this phenomenon, we performed another video urodynamic study. This time, in the filling phase, we noticed the following: 1. the patient’s bladder compliance returned surprisingly to normal (C = 31 ml/cmH_2_O), and 2. leakage of contrast from the bladder to rectum was found on the lateral radiograph. Although a follow-up colonoscopy found no obvious abnormality, cystoscopy and a methylene blue test confirmed the existence of a 1 cm diameter fistula on the posterior wall of the bladder.

**Table 1:** Results of two times urodynamic tests in this patient

	Pdet.max (cmH2O) During Filling phase	Pves.max (cmH2O) During Filling phase	bladder compliance (ml/cmH2O)	DO	Ureteral reflux	detrusor contracts during urination	Qmax	PVR (ml)
1st	102	120	2.12	Yes	No	No	No voluntary voiding	18
2nd	32	64	31	Yes	No	No	No voluntary voiding	16

Qmax: Maximal urinary flow, *Pdet.max* Maximal detrusor pressure, *Pves.max* Maximal vesical pressure, *PVR* Post-void residual

We speculate that this fistula existed initially but was blocked by substances in the bladder, which were washed out during the first urodynamic study, opening the fistula and causing the leakage of contrast around the bladder during the voiding phase. On the other hand, the opening of the fistula led to a decrease in the internal pressure of the bladder, which made the second bladder compliance result normal.

When the patient learned that she was going to start CIC again after the operation, she rejected our operation proposal to close the fistula. She believed that she had no apparent symptoms other than anal leakage and incontinence and that surgery may make the situation worse. Because of the patient’s attitude, she is currently under close follow-up observation.

## Discussion and conclusions

Most vesicorectal fistulas are caused by intestinal diseases, including diverticulitis, intestinal tumors, and Crohn’s disease [1]. However, obstruction/high internal pressure in the adjacent lumen (e.g., biliary tract) of the intestine is also one of the causes of spontaneous intestinal fistula [2]. The patient had neurogenic bowel and neurogenic bladder. We hypothesized that infection and intravesical and intrarectal distension caused her vesicorectal fistula.

A vesicorectal fistula may induce many complications, including recurrent urinary tract infection, pelvic abscess, peritonitis, and intestinal obstruction. Surgical intervention is usually required, and choosing a single- or two-stage repair depends on the location of the fistula and general health [3]. Non-operative management is a viable option only in selected patients with intravenous total parenteral nutrition, bowel rest, and antibiotics [4].

Therefore, surgical closure of the fistula is undoubtedly necessary. The goal of operative management is to separate and close the involved organs with minimal anatomic disruption and normal long-term function of both systems. However, the greatest problem will be the management of the patient’s bladder after the operation since high intravesical pressure and hydronephrosis will most likely reappear. A mixture of medication/botulinum toxin/surgery will be necessary to achieve a low-pressure storage system. Fistula repair combined with bladder enlargement may be a good option if the patient is willing to start CIC again.

Despite the disadvantages of being expensive and requiring expertise, the advantages of video urodynamic studies in the diagnosis of complex urinary dysfunction are still irreplaceable. In this case, it can be predicted that if we choose to initially give patients a conventional urodynamic study, the final diagnosis will greatly altered.

We believe that this is the first report of spontaneous vesicorectal fistula due to neurogenic bladder and neurogenic bowel. Although it resulted in the patient’s firm rejection of the operation, the delicate balance of the patient at present is also worth discussing. Early repair of the fistula is still necessary. The treatment of neurogenic bladder after fistula repair will be difficult and deserves further observation and follow-up.

## Data Availability

Data sharing is not applicable to this article, as no datasets were generated or analyzed during the current study.

## Abbreviations

CIC: Clean intermittent self-catheterization
Pdet.max: Maximal detrusor pressure
Pves.max: Maximal vesical pressure
PVR: Post-void residual
Qmax: Maximal urinary flow
